# Rational Design of Core–Shell MoS_2_@ZIF-67 Nanocomposites for Enhanced Photocatalytic Degradation of Tetracycline

**DOI:** 10.3390/nano15070545

**Published:** 2025-04-03

**Authors:** Maruthasalam Pannerselvam, Vadivel Siva, Anbazhagan Murugan, Abdul Samad Shameem, Thirugnanam Bavani, Sahadevan Jhelai, Sengottaiyan Shanmugan, Imran Hussain Showkath Ali, Karthik Kannan

**Affiliations:** 1 Department of Physics, Karpagam Academy of Higher Education, Coimbatore 641021, Tamil Nadu, India; pannerselvam@kahedu.edu.in (M.P.); jhelaisahadevan@kahedu.edu.in (S.J.); 2 Centre for Energy and Environment, Karpagam Academy of Higher Education, Coimbatore 641021, Tamil Nadu, India; murugan.a@kce.ac.in (A.M.); shameem.abdulsamad@kahedu.edu.in (A.S.S.); bavani.thirugnanam@kahedu.edu.in (T.B.); 3 Department of Science and Humanities, Karpagam College of Engineering, Coimbatore 641032, Tamil Nadu, India; 4 Department of Science and Humanities, Karpagam Academy of Higher Education, Coimbatore 641021, Tamil Nadu, India; 5 Research Centre for Solar Energy, Integrated Research and Discovery, Department of Physics, Koneru Lakshmaiah Education Foundation, Green Fields, Vaddeswaram, Guntur 522502, Andhra Pradesh, India; shanmugan@kluniversity.in; 6 Centre for Micro Nano Design and Fabrication, Department of Electronics and Communication Engineering, Saveetha Engineering College, Chennai 602105, Tamil Nadu, India; imran.usan@gmail.com; 7 Institute of Agricultural Engineering, Saveetha School of Engineering, Saveetha Institute of Medical and Technical Sciences, Chennai 602105, Tamil Nadu, India

**Keywords:** zeolitic imidazolate framework-67, MoS_2_@ZIF-67, core–shell, tetracycline, photocatalytic degradation

## Abstract

Zeolitic imidazolate frameworks (ZIFs) and their composites are attractive materials for photocatalytic applications due to their distinct characteristics. Core–shell ZIFs have lately emerged as a particularly appealing type of metal–organic frameworks, with improved light-absorption and charge-separation capabilities. In this study, hybrid nanocomposite materials comprising a zeolitic imidazolate framework-67 and molybdenum disulfide (MoS_2_) were fabricated with a core–shell structure. The prepared core–shell MoS_2_@ZIF-67 nanocomposites were studied using XRD, FTIR, XPS, and HR-TEM techniques. The crystalline nature and the presence of characteristic functional groups of the composites were analyzed using XRD and FTIR, respectively. The photocatalytic degradation of antibiotic tetracycline (TC) was measured using visible light irradiation. Compared to pristine MoS_2_ (12%) and ZIF-67 (34%), the most active MoS_2_@ZIF-67 nanocomposite (72%) exhibited a greater tetracycline degradation efficacy.

## 1. Introduction

With the social economy’s ongoing expansion and industrial capacity’s rapid development in recent decades, its associated concerns about the environment are becoming severe. Worldwide, the production of antibiotics has increased significantly in the last few decades [1,2]. Many river ditches have dark, foul-smelling water due to the vast volume of antibiotics and other organic effluent produced by the pharmaceutical sector. This has an immense adverse effect on people’s health [3]. The pollution of the water environment has gained increasing attention. A frequently used broad-spectrum antibiotic in clinical treatment, aquaculture, and other domains is tetracycline hydrochloride (TC). When TC is used excessively, it builds inside of wastewater in significant amounts, endangering both human health and ecosystems [4,5,6]. Finding more efficient methods to break down TC in wastewater is critically needed because of its stable chemical structure and antibacterial qualities, which make it difficult to biodegrade [7]. A variety of effective adsorbents, including metal organic frameworks (MOFs), metal oxides, zeolites, activated carbons, and graphene oxide, have been widely used for removing antibiotics from wastewater [8]. Despite the adsorption method’s superior qualities, a thorough breakdown of organic contaminants is still required. Semiconductor photocatalysis is a viable, economical, and ecologically benign method that holds tremendous promise for addressing energy and environmental pollution problems [9,10].

Because photocatalysis on semiconductor surfaces is effective, inexpensive, and environmentally benign in producing clean, renewable hydrogen and breaking down contaminants, it has garnered a lot of interest. WO_3_, TiO_2_, CdS, and ZnO are only a few of the many photocatalytic inorganic semiconductors that have been investigated [11,12,13]. They share several benefits, such as a visible response, abundance, and suitable bandgap and band location. However, because of their low specific surface areas, limited visible light harvesting capacity, simple charge carrier recombination, and non-photo corrosion resistance, pristine inorganic semiconductors continue to have inadequate photocatalytic performance [14,15]. The invention of excellent visible-light-driven photocatalysts is critical to their successful use. In general, photocatalyst systems must possess distinct properties to initiate a photocatalytic reaction, such as a bandgap energy that corresponds to the visible light spectrum (i.e., a high response to visible light), effective separation and migration of electron–hole pairs to prevent charge recombination, and appropriate redox potential [16,17,18]. Using two or more semiconductors in a hybrid photocatalytic heterostructure has been a popular strategy for meeting the conditions, resulting in increased photocatalytic activity.

Noble-metal-free molybdenum disulfide has fascinated researchers due to its extraordinary properties, including strong oxidizing activity, excellent stability and hardness, non-toxicity, huge surface area, and a high proportion of catalytically active sites. Some of the most common applications for MoS_2_ include photocatalytic hydrogen evolution, oxidative desulfurization, and the photocatalytic destruction of organic contaminants [19,20,21]. Furthermore, MoS_2_ has a high adsorption capacity and a customizable band structure. Based on the advantages listed above, MoS_2_ has been widely used as a co-photocatalyst in the creation of composites, particularly in the field of photocatalysis for the removal of organic pollutants. To improve the organic wastewater treatment strategy and more thoroughly investigate the use of MoS_2_ in the field of photocatalytic harm of organic materials [22,23].

Numerous organic materials have been developed as photocatalysts in addition to inorganic semiconductors. Because of their high specific surface area, tunable surface, and adjustable pore size, metal organic frameworks (MOFs), which are made up of metal clusters and organic connectors, have been extensively studied as promising photocatalysts [24,25]. However, pure MOFs are not good photocatalysts because of their high recombination rate of photo-generated electron–hole pairs, high electrical resistance, low photoresponsivity, and other characteristics [26]. A technique of creating heterogeneous structures between MOFs and inorganic semiconductors has been developed to ignite their photocatalytic activity under visible light or sunshine to overcome these drawbacks and fully utilize the benefits [27]. The fabrication of MOFs as efficient solid materials is regarded as one of the most exciting developments in contemporary material science because of their many significant catalytic and photocatalytic characteristics. Zeolitic imidazolate frameworks, a new subclass of MOFs made up of tetrahedral coordinated transition-metal cations (Zn, Co cations) and imidazolate linkers, have recently gained prominence in heterogeneous catalysis due to their abundant functionalities, rapid electron transfer ability, and exceptional chemical and thermal stabilities [28,29]. In recent years, zeolitic imidazolate framework (ZIF) based materials have gained significant attention as photocatalysts for hydrogen evolution and pollution remediation. Notable examples include ZIF-8, B–TiO_2−x_@ZIF-67, and ZIF-8/Fe_2_O_3_ [30,31,32]. Among various ZIF compounds, ZIF derived cobalt (Co) and zinc (Zn) nanoparticles have proven to be highly effective hydrogenation catalysts, particularly for CO_2_ reduction and the selective hydrogenation of unsaturated hydrocarbons. Their exceptionally high surface area enhances active site exposure, while the confinement effect within their porous structure facilitates efficient reactant diffusion. Furthermore, synergistic interactions with co-catalysts, such as MoS_2_, TiO_2_, and Fe_2_O_3_, further enhance their catalytic activity. One of the most promising heterogeneous photocatalysts is ZIF-67, which consists of imidazolate ligands and cobalt cations. It possesses a uniform pore structure, a large surface area, and a distinctive morphology, making it highly suitable for catalytic applications. These characteristics contribute to its superior performance in photocatalytic reactions, particularly in environmental remediation and sustainable energy applications.

**Figure 1:**
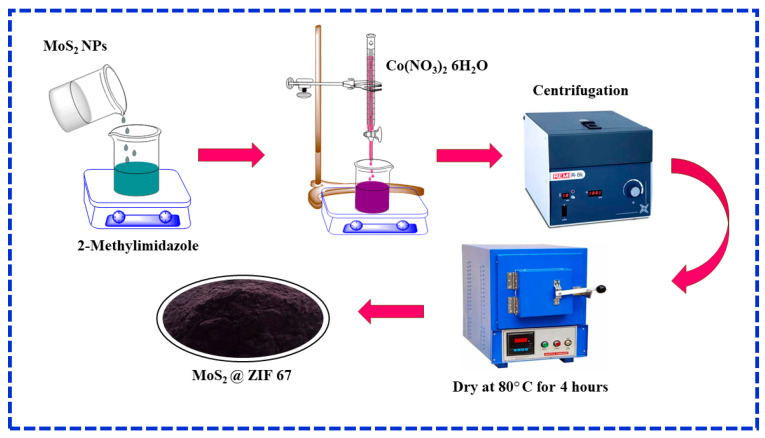
Schematic illustration for the synthesis of MoS_2_@ZIF-67 hybrid nanocomposite.

Additionally, the cage-like structure of ZIF-67 makes it easier to create hollow and core–shell structures through recombination with other semiconductor materials [33,34]. As a result, ZIF-67 is widely used in the photocatalysis industry. The sandwich structure of alternating sulfur and molybdenum layers, or the distinctive “sandwich” structure of MoS_2_, garnered a lot of attention [35]. Based on these factors, the combination of MoS_2_ with ZIF-67 may result in increased photocatalytic activity and stability compared to the individual constituents. The MoS_2_@ZIF-67 heterojunction interface can facilitate the separation and transfer of photogenerated charge carriers, resulting in more efficient photocatalytic processes. In this study, we synthesized and characterized a new MoS_2_@ZIF-67 hybrid for the degradation of tetracycline hydrochloride under visible light (Figure 1).

## 2. Materials and Methods

### 2.1. Materials

AR grade of 2-methylimidazole (C_4_H_6_N_2_), cobaltous nitrate hexahydrate (Co(NO_3_)_2_·6H_2_O), and molybdenum disulfide (MoS_2_) were purchased from Sigma Aldrich, St. Louis, MO, USA. Methanol was used as a solvent for preparation of ZIF-67 and MoS_2_@ZIF-67 nanocomposite.

### 2.2. Preparation of Core–Shell MoS_2_@ZIF-67 Composites

According to earlier reports, the nanocrystals of ZIF-67 were prepared using *in situ* chemical method [36,37]. 2-Methylimidazole and cobalt nitrate hexahydrate were dissolved in 50 mL of methanolic solution. The C_4_H_6_N_2_ was treated with 0.4 g of MoS_2_ before adding a drop-by-drop solution of Co(NO_3_)_2_·6H_2_O and stirring for 2 h. The final solutions were carefully mixed and stirred at room temperature for 4 h, and the mixed materials were kept in 18 h. After, the precipitate was washed with methanol several times (to remove unreacted residues). Finally, the obtained MoS_2_@ZIF-67 nanocomposite was dried in 80 °C for 4 h in vacuum oven.

### 2.3. Photocatalytic Degradation Analysis

Photocatalytic degradation activity of the prepared photocatalysts was analyzed based on the degradation of the tetracycline hydrochloride (TC) under visible light. For this experiment, 100 mg of the catalyst was added in the TC (6 × 10^−5^ M) solution containing a vessel placed in the photocatalytic chamber with 160 W of tungsten halogen lamp as visible light source. To attain the sorption equilibrium, the solution was stirred for 30 min under dark circumstance, before irradiation of light. After that, 5 mL of aliquots was collected at regular time intervals under illumination of light. Then, the excess of catalyst was removed using centrifugation to further analyze its concentration at absorption maximum (λ) at 357 nm by using UV-vis spectrometer.

### 2.4. Band Gap Calculation

A standard relation Kubelka–Munk function was used to calculate the bandgap energy value of the prepared materials (Equation (1)) [38].

αhν = A (hν − E_g_)^n^ (1)

where α, E_g_, ν, h, n, and A signify the absorption coefficient, band gap energy, frequency of light, Planck’s constant, nature of the photocatalyst (n = 1/2 for direct bandgap semiconductors and n = 2 for indirect bandgap semiconductors), and proportionality constant, respectively.

### 2.5. Band Potential Calculation

The conduction band (CB) and valence band (VB) potentials are essential parameters for understanding the electronic structure and photocatalytic behavior of semiconductors. These potentials can be estimated using the following equations (Equations (2) and (3)) [39,40],

E_VB_ = X − E^e^ + 0.5E_g_ (2)

E_CB_ = E_VB_ − E_g_ (3)

where E_CB_ and E_VB_ are the potentials of conduction and valence band, respectively, X represents the absolute electronegativity of the semiconductor, E^e^ is the energy of free electrons on the hydrogen scale (~4.5 eV), and E_g_ is the bandgap energy value of the semiconductors. This equation determines the VB and CB potential based on the material’s electronegativity and bandgap energy.

### 2.6. Characterization Details

The structural information was analyzed using Bruker D8 ECO advanced X-ray diffraction (Karlsruhe, Germany) with Cu Kα radiation (λ = 1.54 Å). JEOL JEM 2100 (Tokyo, Japan) Plus high-resolution transmission electron microscopy (HRTEM) was used to confirm the core–shell structure of the nanocomposites. The CHI660 F electrochemical workstation (Shanghai, China) was utilized to analyze the electrochemical properties using 3 electrode system in 1 mol KOH. Shimadzu IR tracer–100 (Kyoto, Japan) was used to confirm the vibrations of pure and composites in the range from 4000 to 400 cm^−1^. The chemical stability and composition were evaluated using Omicron ESCA, made by Oxford Instruments, Wiesbaden, Germany. The optical properties were studied with UV–Visible spectroscopy (JASCO V750, Tokyo, Japan) in the range of 200–800 nm.

## 3. Results and Discussion

### 3.1. Structural and Surface Morphology

**Figure 2:**
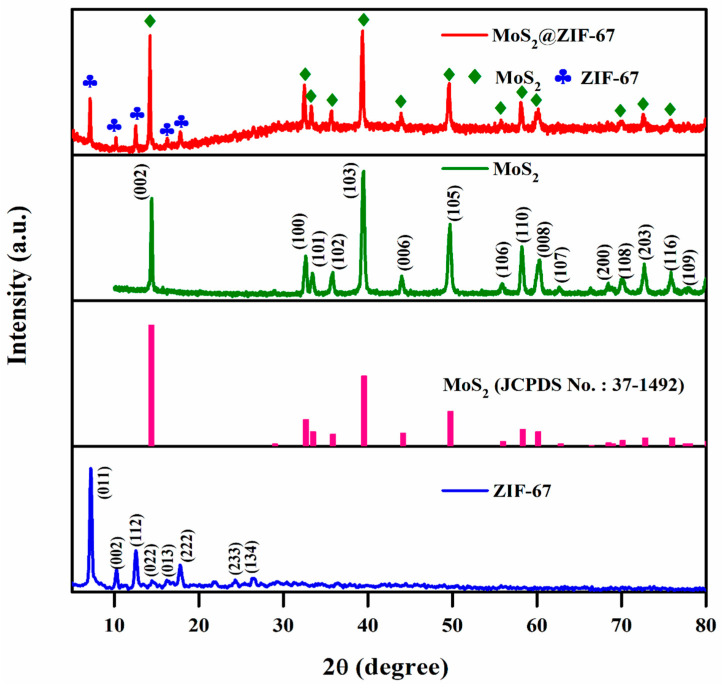
XRD patterns of pure and nanocomposites.

The structural information of the pristine MoS_2_, ZIF-67, and MoS_2_@ZIF-67 nanocomposites was studied using XRD, and the patterns are shown in Figure 2. The nanocomposite displayed peaks at 2θ values of 7.31° (0 1 1), 10.35° (0 0 2), 12.72° (1 1 2), 14.41° (0 2 2), 16.46° (0 1 3), 18.03° (2 2 2), 24.54° (2 3 3), and 26.72° (1 3 4), which match the crystalline structure of ZIF-67 and are in good agreement with previous reports [33,41]. Furthermore, high-intensity peaks are presented at 14.28° (0 2 2), 32.46° (1 0 0), 39.45° (1 0 3), 49.84° (1 0 5), and 57.36° (1 1 0). These planes were identified as MoS_2_ (JCPDS No.: 37-1492), indicating the existence of MoS_2_ in the MoS_2_@ZIF-67 nanocomposite [42]. The MoS_2_@ZIF-67 nanocomposite shows two distinct crystalline phases of ZIF-67 and MoS_2_. The strong and dominating peaks in the nanocomposite’s XRD diffractogram suggest that the MoS_2_ was extremely crystalline and maintained its crystallinity perfectly through the preparation of core–shell MoS_2_@ZIF-67. The average crystallite size of the materials was calculated using the Scherrer relation, and the values are MoS_2_ (49 nm), ZIF-67 (35 nm), and MoS_2_@ZIF-67 (40 nm).

**Figure 3:**
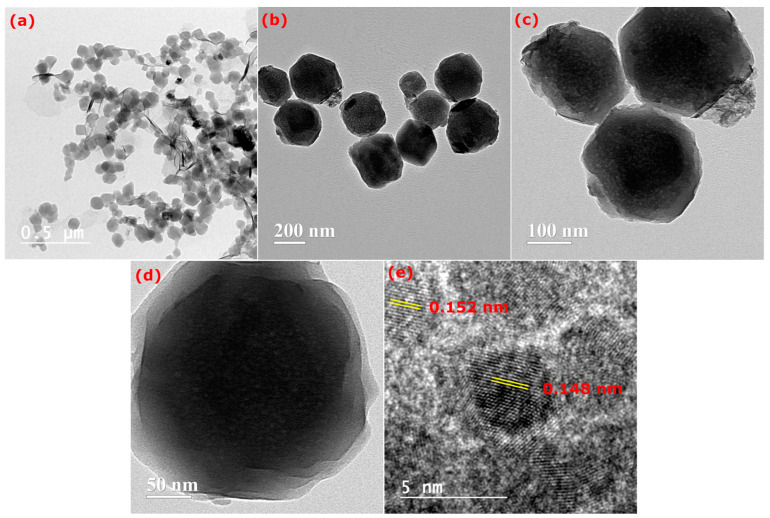
HR-TEM image of (**a**) ZIF-67; (**b**–**d**) MoS_2_@ZIF-67 with different resolutions; and (**e**) lattice fringes with d-spacings of ZIF-67.

HR-TEM images of the pristine ZIF-67 are presented in Figure 3a. Figure 3b–d present HR-TEM images of MoS_2_@ZIF-67, illustrating the irregular polygonal structures of ZIF-67 and the non-uniform nanosheets of MoS_2_, respectively. Additionally, Figure 3c shows that the MoS_2_ nanosheets are irregularly encapsulated on the ZIF-67 framework [43]. Figure 3d distinctly illustrates the core–shell architecture with polyhedral morphology, signifying the encapsulation of MoS_2_ nanoparticles (shell) around the core of ZIF-67. Figure 3e displays HRTEM images that exhibit lattice fringes with d-spacings of 0.152 nm and 0.148 nm, as indicated in the Figure (ZIF-67).

### 3.2. FT-IR Analysis

**Figure 4:**
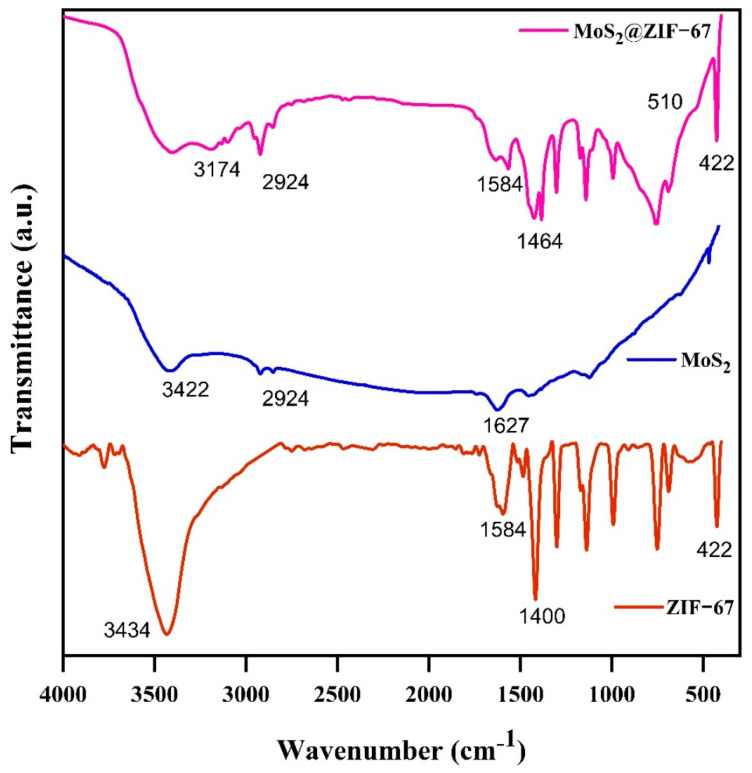
FT-IR spectra of pure MoS_2_ and ZIF-67, and nanocomposites.

The functional group of materials was investigated using FT-IR in the wavenumber region of 4000–400 cm^−1^. The FT-IR spectra of MoS_2_, ZIF-67, and MoS_2_@ZIF-67 are displayed in Figure 4. The band of 3300–3500 cm^−1^ corresponds to the stretching vibration of O-H bonds in adsorbed water molecules [44]. Stretching vibrations at 1418 cm^−1^ and 1584 cm^−1^, attributed to the aromatic imidazole ring, are also observed in the spectra. Peaks occurring between 600 and 1500 cm^−1^ were associated with the aromatic bending and stretching vibrational modes. Additionally, the imidazole group exhibited out-of-plane bending vibrations at 751 cm^−1^, while in-plane bending vibrations are observed in the range of 1000–1300 cm^−1^ [43]. The same vibration is present in pure MoS_2_, ZIF-67, and MoS_2_@ZIF-67. The broad band at 3174 cm^−1^ is due to the C-H stretching vibrations of the methyl group in the 2-methylimidazole linker. The medium peak at 1584 cm^−1^ indicates the C=N stretching mode of 2-Methylimidazole [33]. The bands in the 1400–600 cm^−1^ region correspond to the stretching and bending modes of the imidazole ring. Most observed vibrational bands are related to the vibrations of imidazole, except for the Zn-N stretching at 422 cm^−1^ [37]. In addition, the peak at about 1464 cm^−1^ in the nanocomposite belongs to the S-Mo-S bond and a small peak at 510 cm^−1^ is due to the S–S bond [45]. From the FTIR analysis, all the characteristic vibrations of ZIF-67 and MoS_2_ were presented in the composite and confirm the formation of MoS_2_@ZIF-67.

### 3.3. X-Ray Photoelectron Spectral Analysis

**Figure 5:**
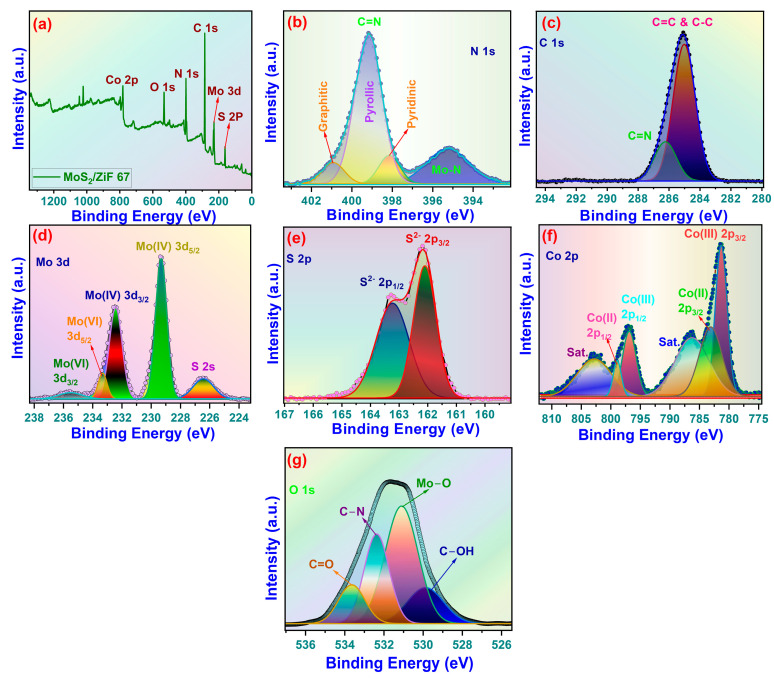
(**a**) Survey spectrum of MoS_2_@ZIF-67, and core-level spectra of (**b**) N 1s, (**c**) C 1s, (**d**) Mo 3d, (**e**) S 2p, (**f**) Co 2p of MoS_2_@ZIF-67, and (**g**) O 1s of MoS_2_@ZIF-67.

To further substantiate the effective recombination of MoS_2_ and ZIF-67, we examined the elemental valence state of the sample. Figure 5a illustrates the XPS spectrum of the MoS_2_@ZIF-67 composite. Figure 5 shows that N, C, Co, Mo, O, and S were found in the full XPS spectrum of the composite material and the XPS content is shown in Table 1.

In Figure 5, the high-resolution N 1s spectrum shows peaks at 400.7 eV, 399.2 eV, and 395.2 eV, which are N=N, imidazole-N, and N^3−^, respectively, along with a small satellite peak. Except for the imidazole-N coordination bond from the ZIF-67 material, the previous peak displays a comparatively greater bonding energy than the others [46,47]. The amount of three nitrogen species was assessed by fitting the area to the nitrogen 1s curve. The proportion of each nitrogen functional group can be quantified using the XPS method, which detects the binding energy of chemical bonds. The binding energies of four nitrogen functional groups are as follows: pyrrolic N–H at 400 eV; pyridinic N at 398.4 eV; graphitic N at 401 eV; and pyridinic N–O at 402 and 404 eV [48,49]. The percentage of deconvoluted peaks is shown in Table 1. In total, 22% of the peaks were at 395.2 eV, 66% at 399.1 eV, and 12% 400.6 eV. The results indicate that the pyrolysis peak was diminished, the overall nitrogen content and pyridine nitrogen content was higher, but the graphite nitrogen concentration diminished [50,51]. The C 1s spectra (Figure 5c) were deconvoluted into two peaks: one at 284.8 eV attributed to the C=C bond, and another at 286 eV associated with the C-N bond [52]. Figure 5d illustrates the high-resolution Mo 3d spectra of MoS_2_@ZIF-67 composites. The distinct peaks at 228.9, 230.9, 232.5, and 235.4 eV are attributed to Mo^4+^ 3d_3/2_, Mo^4+^ 3d_5/2_, and Mo^6+^ 3d_5/2_, and Mo^6+^ 3d_3/2_, respectively [53,54]. This implies that Mo ions in the composite material coexist as Mo^4+^ and Mo^6+,^ with the peak of Mo^6+^ being less pronounced than that of Mo^4+^, signifying a lower concentration of Mo^6+^. The simultaneous presence and transformation of Mo^4+^ and Mo^6+^ can enhance electron transport during the degradation process, thereby facilitating the generation of active compounds [55]. The doublet separation of Mo 3d_5/2_ and 3d_3/2_ is approximately 3.1 eV, which is in good agreement with Amin et al. [56]. We detected a minor peak at a binding energy of 226.3 eV, which corresponds to the S 2s spectrum [57]. The high-resolution S 2p spectra of the composite material are presented in Figure 5e, with two prominent peaks at 161.9 eV and 163.1 eV, corresponding to S^2−^ 2p_3/2_ and S^2−^ 2p_1/2_, respectively [58,59]. The findings indicate that a significant quantity of unsaturated sulfur in the composites enhances photo/electrocatalytic activity. The Co 2p spectrum of MoS_2_@ZIF 67 (Figure 5f) displays primary peaks at 781.8 eV and 797.4 eV, respectively, signifying the Co (II) oxidation state. Furthermore, we attribute the peaks observed at 782.8 eV and 798.5 eV to Co 2p_3/2_ and Co 2p_1/2_, respectively, indicating a Co (III) oxidation state. The binding energies of 786.5 eV and 802.8 eV correspond to the conventional Co 2p3/2 and Co 2p1/2 satellite peaks, respectively [60,61]. The doublet separation of Co 2p_3/2_ and 2p_1/2_ is approximately 15 eV [62]. The XPS survey revealed a small O1s peak at 531.5, indicative of C–O [63,64]. This signal may indicate the presence of chemisorbed oxygen or other hydroxyl-containing molecules [65]. The lack of a signal at 496.5 eV may result from surface oxidation in the sample exposed to the environment [66]. The presence of oxygen-containing functional groups may enhance the electrical conductivity and surface wettability in the composites, hence facilitating superior degradability. Figure 5g demonstrates that the O 1 s XPS signal can be deconvoluted into four peaks at 530.2, 531.4, 532.4, and 533.3 eV, which correspond to C=O, C–O, Mo–O, and C–OH bonds, respectively [67,68]. A chemical bond between molybdenum and oxygen promotes the attachment of paramolybdate ions to the ZIF-67 surface, leading to the formation of MoS_2_ nanosheets. This is corroborated by a study utilizing a transmission electron microscope.

### 3.4. Optical Absorption Studies

**Figure 6:**
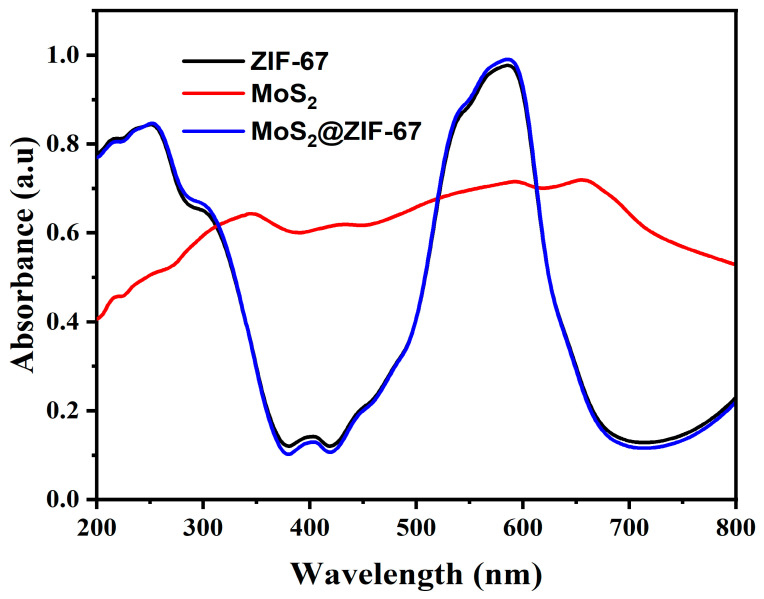
DRS UV-Vis of ZIF-67, MoS_2_, and MoS_2_@ZIF-67 hybrid photocatalysts.

**Figure 7:**
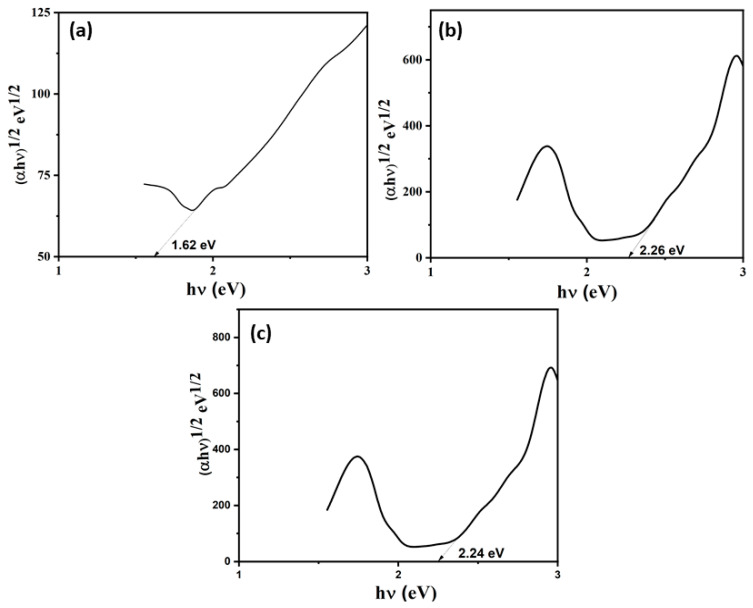
Kubelka–Munk plots of (**a**) MoS_2_, (**b**)ZIF-67, and (**c**) MoS_2_@ZIF-67 hybrid photocatalysts.

The optical absorption properties of the photocatalysts were examined using a UV–visible diffuse reflectance spectral (DRS) study. Figure 6 illustrates the absorption spectra of the pure ZIF-67, MoS_2_, and MoS_2_@ZIF-67 hybrid. Herein, the absorption of ZIF-67 was observed at 252 and 585 nm, MoS_2_ at 656 nm, and MoS_2_@ZIF-67 composite at 251 and 584 nm, respectively. The shoulder peaks observed in the ZIF-67 are for the ligand metal charge transfer transition, while the strong absorption is due to the 4A2 (F) → 4T1 (P) transition of Co^2+^ of ZIF-67. The calculated band gap energy results for the pure ZIF-67 and MoS_2_, and the MoS_2_@ZIF-67 hybrid of 2.26, 1.62, and 2.24 eV, respectively, are shown in Figure 7. The prepared MoS_2_@ZIF-67 hybrid has a lower bandgap energy value when compared with pure ZIF-67, representing the greater absorption ability of the hybrid [43,69].

**Figure 8:**
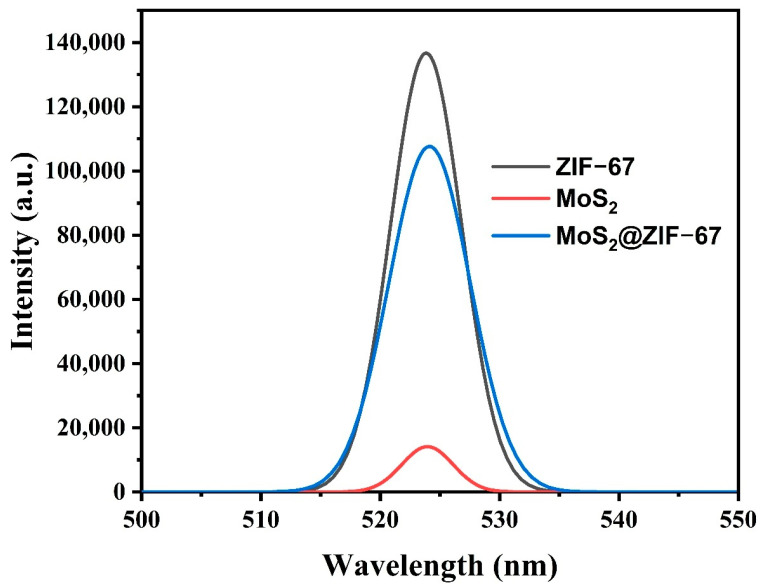
PL spectra of ZIF-67, MoS_2_, and MoS_2_@ZIF-67 hybrid photocatalysts.

To the addition of the bandgap of the photocatalyst, the rate of reconnection for the photoinduced charge carriers also influences the photocatalytic capability; hence, the photoluminescence spectra was investigated for ZIF-67, MoS_2_, and MoS_2_@ZIF-67 hybrid photocatalysts under a 320 nm excitation wavelength. The results are shown in Figure 8. In general, the higher PL peak intensity suggests the lower carrier’s separation and migration. As seen in Figure 8, the greater peak intensity of ZIF-67 implies the greater reconnection of charges in the ZIF-67; at the same time, the pure MoS_2_ shows a lower PL intensity. In the case of the MoS_2_@ZIF-67 hybrid, the PL emission intensity was lower than the pure ZIF-67 due to its formation of a hybrid between ZIF-67 and MoS_2_, which signified effective separation and migration of the charge with an increased life span [70,71].

### 3.5. Photocatalytic Performance Analysis

**Figure 9:**
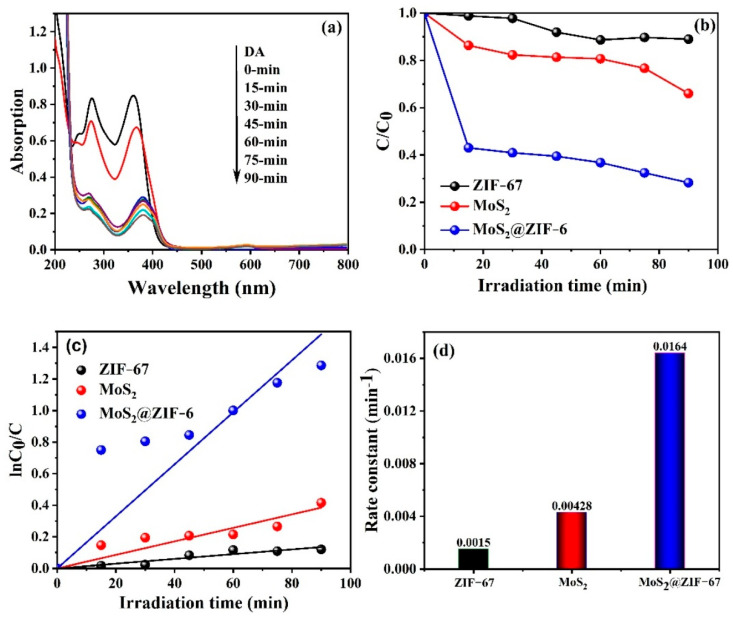
(**a**) UV-vis spectra for the degradation of TC under visible light irradiation over MoS_2_@ZIF-67 hybrid. (**b**) Photocatalytic degradation, (**c**) Pseudo-first-order kinetics plots and (**d**) bar diagram for the rate constant values of pure ZIF-67 and MoS_2_, and MoS_2_@ZIF-67 hybrid.

The photocatalytic abilities of the fabricated materials were evaluated using the degradation of TC under simulated light illumination for 90 min. Figure 9a depicts the UV-vis absorption spectra for the MoS_2_@ZIF-67 hybrid towards the degradation of the TC under visible-light irradiation. This shows that the distinctive peak at maximum absorption of 357 nm steadily decreases with an increasing illumination time, resulting in the effective disappearance of the peak during the degradation of TC. Furthermore, the photocatalytic degradation efficiency of the pure ZIF-67 and MoS_2_, and the MoS_2_@ZIF-67 hybrid photocatalysts was 34, 12, and 72% respectively; the results are illustrated in Figure 9b. The MoS_2_@ZIF-67 hybrid revealed substantial enhancement in the degradation of TC within 90 min of irradiation, accredited to the effective formation of heterojunction that assists the quick separation and transfer of photoproduced carriers. The kinetics for the degradation of TC were analyzed by using the pseudo-first-order kinetics equation given in Equation (4) [43,72],

lnC_t_/C_0_ = kt (4)

where C_t_ and C_0_ are the concentration of the dye at different time intervals and initially, respectively, k and t are the pseudo-first-order rate constant (min^−1^) and time (min), respectively. As seen in Figure 9c,d, the kinetic rate constant value of the pure ZIF-67 and MoS_2_, and the MoS_2_@ZIF-67 hybrid was found to be 0.0015, 0.00428, and 0.0164 min^−1^, respectively. The above results underscore the greater photocatalytic activity of the MoS_2_@ZIF-67 hybrid, indicating the effective formation of the heterojunction between the MoS_2_ nanoparticle and ZIF-67, which possibly encourages effective absorption in the visible-light region and improves the lifespan of the charges.

**Figure 10:**
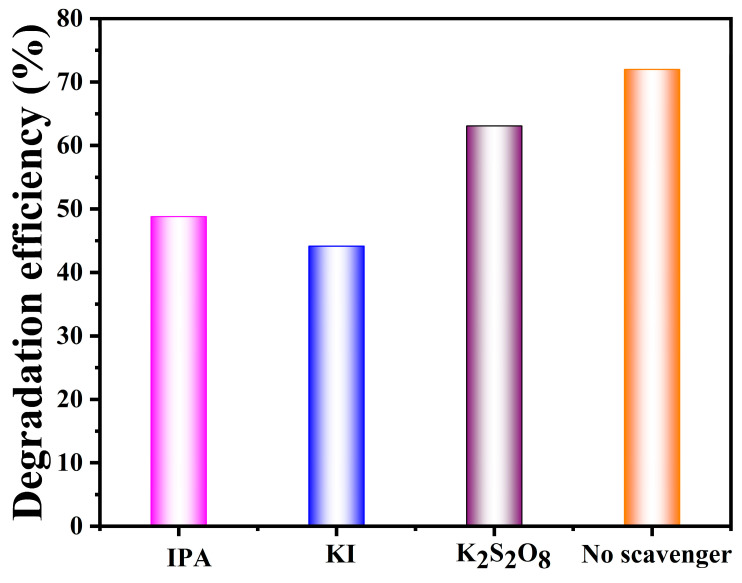
Effect of radical quenchers in the degradation of TC over MoS_2_@ZIF-67 under visible-light irradiation.

Table 2 compares ZIF-67- and MoS_2_-based composites for antibiotic degradation. ZIF-67 composites offer a high surface area, tunable porosity, and strong catalytic properties, enhancing the degradation efficiency. In contrast, MoS_2_ composites excel in photocatalytic degradation, which is influenced by the light frequency and material morphology. Both materials show promising results in antibiotic removal due to their edge-active sites and capacity to create ROS under UV light [73]. Depending on the catalytic mechanism, ZIF-67 composites degrade a wide range of antibiotics, such as tetracycline and ciprofloxacin, efficiently in both UV and visible environments. ZIF-67’s high surface area and porosity increase the contact area between antibiotics and catalysts, which improves the degradation process. The selection of MoS_2_@ZIF-67 composites for a given application is determined by the needed reaction conditions, stability, and degradation efficiency for tetracycline. As illustrated in Figure 10, radical scavenging was used to determine the activity of the key radical in the TC degradation over the MoS_2_@ZIF-67 hybrid, by adding various quenchers, such as isopropanol (IPA), potassium iodide (KI), and potassium persulfate (K_2_S_2_O_8_), at a concentration of 1 mmol L^−1^ to quench the hydroxyl (OH^●^), holes (h^+^) and electrons (e^−^), respectively. A sudden decrease in the photocatalytic activity was observed following the addition of KI and IPA, a scavenger of h^+^ and OH. radicals. The addition of the K_2_S_2_O_8_ scavenger of e^−^ exhibited a negligible impact on the degradation of TC. These results revealed that the h^+^ and OH. radicals are majorly active radicals in the degradation of TC over the MoS_2_@ZIF-67 hybrid.

**Figure 11:**
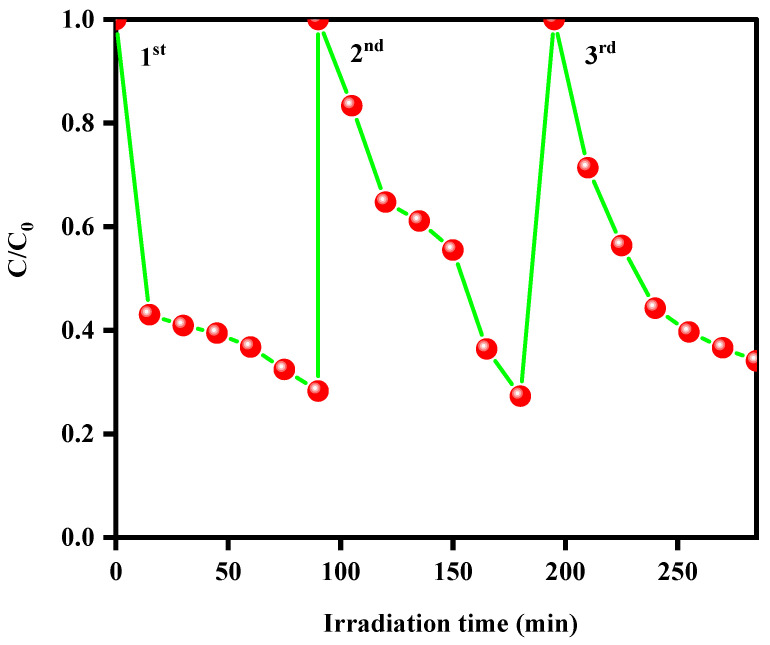
Recycling runs of MoS_2_@ZIF-67 hybrid over the degradation of TC under visible light irradiation.

Additionally, the recyclability of the photocatalyst is a crucial factor for practical applications, which determines their long-term reusability and cost-effectiveness. Figure 11 exposes the recycling runs of the MoS_2_@ZIF-67 hybrid over the degradation of TC. For the experiment, the MoS_2_@ZIF-67 hybrid photocatalyst was subjected to three consecutive degradation cycles, with the degradation rate monitored after each cycle. From Figure 10, negligible changes were observed even after three cycles. This demonstrates the excellent photostability and recyclability of the MoS_2_@ZIF-67 hybrid.

Table 3 shows a comparison of the radical’s activity experiments of MoS_2_ and ZIF-67 degradation of tetracycline. Photocatalytic radical trapping experiments are essential for identifying and understanding the reactive species involved in photocatalytic processes. These experiments typically employ specific scavengers to selectively quench reactive oxygen species (ROS), such as hydroxyl radicals (OH^−^), superoxide radicals (O_2−_), and photogenerated holes (h^+^), by observing changes in photocatalytic activity upon the introduction of these scavengers.

### 3.6. Electrochemical Performance

**Figure 12:**
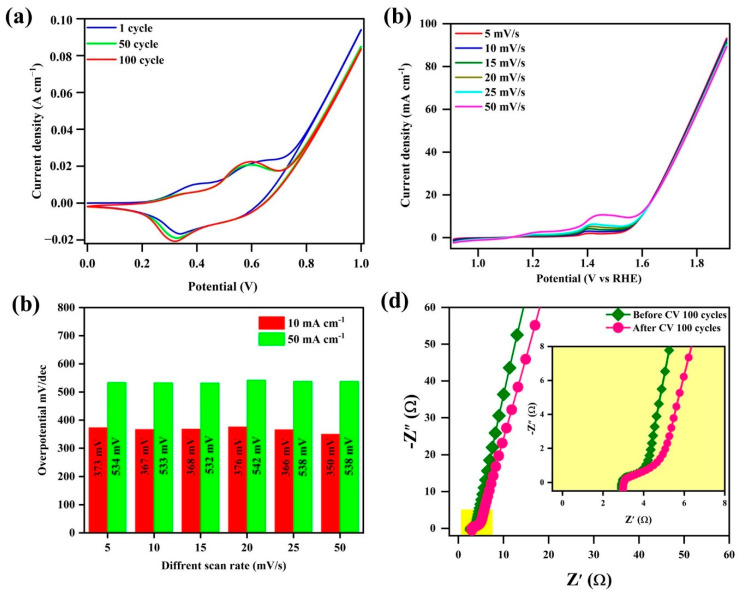
(**a**) CV curves, (**b**) LSV curves, (**c**) bar diagram of LSV potential, and (**d**) EIS plot.

In this study, cyclic voltammetry (CV) was performed to investigate the electrochemical performance of the MoS_2_@ZIF-67 hybrid nanocomposites; the results are shown in Figure 12a. the prepared electrode was examined in a 1 M KOH alkaline electrolyte and examined in a three-electrode configuration at a CHI 660F electrochemical workstation. We prepared the electrode material, Pt wire, and Hg/HgO for the working, counter, and reference electrode, respectively. This technique provides insights into the charge transfer mechanisms and the interaction of the electrocatalyst with the electrolyte [40]. The CV was conducted at a scan rate of 100 mV/s and ran continuously for 100 cycles, optimized to evaluate the reaction kinetics. With an increasing number of cycles, the peak current showed a proportional increase, suggesting a diffusion-controlled charge transfer process [33]. The electrocatalyst material was more stable, with a slight difference between the area of the 1st and 100th cycles, which denotes the excellent material stability. To analyze the role of oxygen and water molecules, moreover, the Linear Sweep Voltammetry (LSV) technique is used for the electrocatalyst of MoS_2_@ZIF67 nanocomposites and the results are shown in Figure 12b. It clearly shows an enhanced catalytic performance, with a lower overpotential of 373 mV at the current density of 10 mA/cm^2^ and 534 mV at the current density of 50 mA/cm^2^ at a scan rate of 5 mV/s.

The LSV analysis at various scan rates, such as 10, 15, 20, 25, and 50 mV/s, is shown in Figure 12c. Generally, in an oxygen evolution reaction, OH^−^ ions from the electrolyte adsorb onto the active sites of the catalyst, initiating the oxidation process [76]. Water molecules can also act as reactants, undergoing oxidation to form hydroxyl radicals and molecular oxygen [77]. The catalyst facilitates faster charge transfer, as evident from the LSV results at different scan rates (10–50 mV/s), showing variations in the current response and overpotential. The improved catalytic efficiency suggests strong interactions between the MoS_2_@ZIF-67 active sites and the OH^−^/H_2_O species, reducing the energy barrier for oxygen evolution [77], which was confirmed with the obtained lower overpotentials. The bar diagram (Figure 12c) further illustrates how different scan rates influence the current density, confirming the catalyst’s stability and efficiency under varied electrochemical conditions. In Figure 12d, EIS was used to measure the response of the system to a small AC perturbation over a wide frequency range [78].

The prepared electrocatalytic materials were examined using electrochemical impedance spectroscopy (EIS) in a 1 M KOH aqueous solution at a frequency range of 1 to 100 kHz with an applied amplitude potential of 5 mV. A Nyquist plot, where the real part (Z′) corresponds to the charge transfer resistance, and the imaginary part (Z″) reflects the capacitive behavior of the electrode [73], is shown in Figure 12d. In the high- to mid-frequency region, a small semicircle is observed, which also confirmed that the electrocatalytic material exhibited a significantly lower charge transfer resistance, unchanged even after the cycles. This indicates that for the MoS_2_@ZIF-67 photocatalyst, electrons have more difficulty reaching the semiconductor material/electrolyte interface. When photocatalytic degradation occurs, there is a possibility for electrons to recombine before reacting with pollutants. The small semicircle indicated that, during the photocatalytic performance of the materials, there is a recombination of e^−^/h^+^ [79]. These results point out that in the MoS_2_@ZIF-67 photocatalyst, material was degraded to contaminants in the surplus water.

### 3.7. Charge Transfer Mechanism for Degradation of Tetracycline

**Figure 13:**
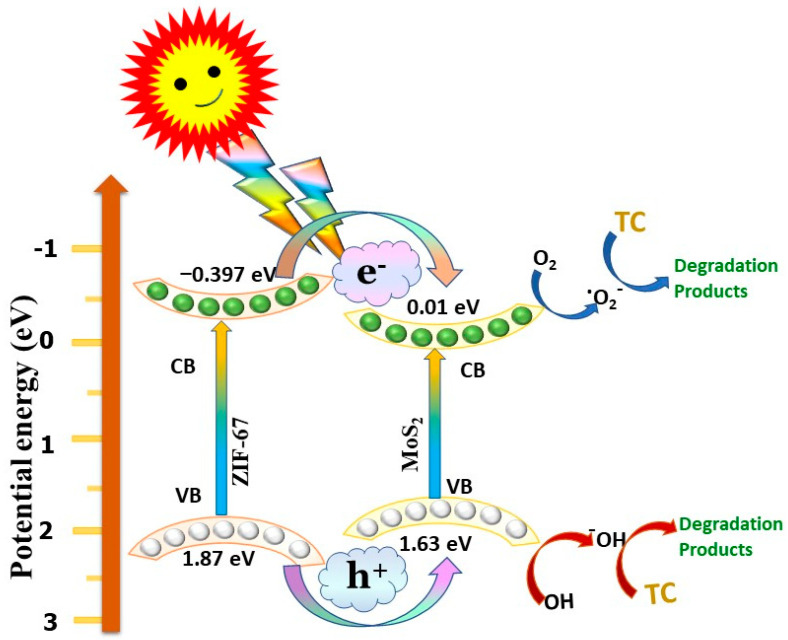
Pictorial illustrations for the charge transfer mechanism of the MoS_2_@ZIF-67 hybrid towards the degradation of TC.

A possible type-I heterojunction charge transfer mechanism was proposed for the ZIF-67/MoS_2_ hybrid, as shown in Figure 13. Under visible-light illumination, e^−^ in the VB of both ZIF-67 and MoS_2_ are excited from their respective VB into CB. At the same time, the e^−^ in the CB of ZIF-67 migrates into the CB of MoS_2_, due to the more negative potential of ZIF-67. Simultaneously, the h^+^ in the VB of ZIF-67 shifted towards the VB of MoS_2_. The accumulated e^−^ and h^+^ in the CB and VB of MoS_2_ facilitates the reduction and oxidation of surface-adsorbed oxygen and water molecules, converting them into harmless, biodegradable by-products [80,81]. In this way, the charge carriers are separated and their lifespan is extended, which improves the photocatalytic activity by reducing the rapid recombination of charges. The construction of an MoS_2_@ZIF-67 heterojunction accelerates the transfer and separation of charges through the heterojunction and uphold its redox ability for the degradation of TC under visible-light illumination. Combining some of the possible reactions participating in TC degradation of MoS_2_ with ZIF-67 enhances the photocatalytic degradation of tetracycline under visible light. The synergy between these materials facilitates efficient charge separation and increases the generation of reactive oxygen species, leading to an improved degradation efficiency. This approach offers a promising strategy for addressing antibiotic contamination in water sources [69,74,82].

MoS_2_ + hv → MoS_2_ (e^−^) + MoS_2_ (h^+^) (5)

ZIF67 + hv → ZIF-67 (e^−^) + ZIF-67 (h^+^) (6)

O_2_ + g-ZIF-67 (e^−^) → ZIF-67+ O_2_^−^ (7)

H_2_O + MoS_2_ (h^+^) → MoS_2_ + OH^−^ (8)

OH^−^, and O_2_^−^ + TC → degradation products (9)

## 4. Conclusions

Novel core–shell MoS_2_@ZIF-67 nanocomposites were synthesized using a simple *in situ* chemical method, and their characteristics were studied using XRD, FTIR, XPS, PL, and HRTEM. The Co 2p spectrum of MoS_2_@ZIF 67 displayed primary peaks at 781.8 eV and 797.4 eV, respectively, signifying a Co (II) oxidation state. HRTEM analysis suggested that the nanoscale MoS_2_ uniformly grown on the surface of ZIF-67 confirms the core–shell assembly. The band gap energy of MoS_2_@ZIF-67 was found to be 2.24 eV. Moreover, the PL emission intensity of the MoS_2_@ZIF-67 was lower than that of pure ZIF-67; this is due to the formation of a hybrid between ZIF-67 and MoS_2_, which signified the effective separation and migration of the charge with an increased life span. The MoS_2_@ZIF-67 hybrid revealed a substantial enhancement in the degradation of TC within 90 min of irradiation. The kinetic rate constant was evaluated, and the values of pure ZIF-67, MoS_2_, and the MoS_2_@ZIF-67 hybrid were found to be 0.0015, 0.00428, and 0.0164 min^−1^, respectively. These results revealed that the h^+^ and OH. radicals are majorly active radicals in the degradation of TC. An LSV study clearly showed the enhanced catalytic performance, with a lower overpotential of 373 mV at a current density of 10 mA/cm^2^ and 534 mV at a current density of 50 mA/cm^2^ and a scan rate of 5 mV/s.

## Figures and Tables

**Table 1:** Elements and their atomic percentages in composites.

S. No	Element	Atomic %
1.	S 2p	5.67
2.	Mo 3d	2.47
3.	C 1s	62.32
4.	N 1s	21.04
5.	Co 2p	4.11
6.	O 1s	4.39

**Table 2:** Comparative analysis of MoS_2_ and ZIF-67 nanocomposites for antibiotic degradation.

S. No	Photocatalyst	Pollutants	Photocatalytic Efficiency (%)	Irradiation Time (min)	Light Source	Morphology	Ref.
1.	MoS_2_/Ag/g-C_3_N_4_	Tetracycline	98.9	50	300 W Xe lamp equipped with a UV cutoff filter (λ > 420 nm).	Flower-like shape	[72]
2.	BiOBr/MoS_2_/GO	Tetracycline	98	40	Visible 300 W Xe lamp with a 380 nm cut-off filter was used to simulate visible light source.	Flower-like lamellar clusters	[73]
3.	CoS_2_/MoS_2_@Zeolite	Tetracycline	96.71	160	A 300 W Xe-lamp was used as the visible light source and the UV light was filtered by a filter (420 nm).	Spherical hydrangea-like	[74]
4.	Ag_2_MoO_4_/ZIF-67	Tetracycline	98.2	75	under a LED type visible light 50 W illumination with a cut-off filter (420 nm).	Rhombic-like	[39]
5.	C3N4/N, P CQD/ZIF-67	Ciprofloxacin	98	90	Under Visible Light	-	[40]
6.	ZIF-67/MoS_2_/MWCNT	Tetracycline	96.1	80	Visible light 25 W LED lamp.	Rhombic dodecahedron shape	[69]
7.	MoS_2_/ZnO	Ciprofloxacin	89	120	UV light 250 W metal halide lamp	Rod-shaped ZnO microstructure and flakes of MoS_2_.	[75]
8.	MoS_2_@ZIF-67	Tetracycline	72	90	Visible 160 W tungsten lamb	Polyhedral morphology	Present work

**Table 3:** Comparison of radical’s activity experiments of MoS_2_ and ZIF-67 degradation of tetracycline.

S. No	Photocatalyst	Pollutants	Trapping Agent	Active Radicals	Irradiation Time (min)	Light Source	Ref.
1.	MoS_2_/Ag/g-C_3_N_4_	TC	IPA BQ EDTA-2Na MeOH	BQ (O_2_^−^)	50	300 W Xe lamp equipped with a UV cutoff filter (λ > 420 nm).	[72]
2.	BiOBr/MoS_2_/GO	TC	IPA KI Ascorbic acid	KI (h^+^) Ascorbic acid (O_2_^−^)	40	Visible 300 W Xe lamp with a 380 nm cut-off filter was used to simulate visible light source.	[73]
3.	Ag_2_MoO_4_/ZIF-67	TC	IPA Na_2_C_2_O_4_ BQ AO	BQ (O_2_^−^) AO (h^+^)	75	Under a LED type visible light 50 W illumination with a cut-off filter (420 nm).	[39]
4.	C_3_N_4_/N, P CQD/ZIF-67	TC	TEA IPA BQ	TEA (h^+^) IPA (OH^−^)	90	Under Visible Light	[40]
5.	ZIF-67/MoS_2_/MWCNT	TC	IPA EDTA-2Na BQ	IPA (OH^−^) BQ (O_2_^−^)	80	Visible light 25 W LED lamp.	[69]
6.	MoS_2_@ZIF-67	TC	IPA KI K_2_S_2_O_8_	KI (h^+^) IPA (OH^−^)	90	Visible 160 W tungsten lamb	Present work

TC—Tetracycline; IPA—Isopropyl alcohol; BQ—benzenol; EDTA—Ethylenediamine; MeOH—Methyl alcohol; TEA—triethanolamine.

## Data Availability

Data are contained within the article.
